# Ectomycorrhizal Fungal Strains Facilitate Cd^2+^ Enrichment in a Woody Hyperaccumulator under Co-Existing Stress of Cadmium and Salt

**DOI:** 10.3390/ijms222111651

**Published:** 2021-10-28

**Authors:** Chen Deng, Zhimei Zhu, Jian Liu, Ying Zhang, Yinan Zhang, Dade Yu, Siyuan Hou, Yanli Zhang, Jun Yao, Huilong Zhang, Nan Zhao, Gang Sa, Yuhong Zhang, Xujun Ma, Rui Zhao, Andrea Polle, Shaoliang Chen

**Affiliations:** 1 Beijing Advanced Innovation Center for Tree Breeding by Molecular Design, College of Biological Sciences and Technology, Beijing Forestry University, Beijing 100083, China; ced501@163.com (C.D.); zhimeizhu@163.com (Z.Z.); liujian20170703@163.com (J.L.); zying@bjfu.edu.cn (Y.Z.); xhzyn007@163.com (Y.Z.); dyu@gwdg.de (D.Y.); housiyuan2020@163.com (S.H.); zhangyl@bjfu.edu.cn (Y.Z.); yaojun990@126.com (J.Y.); zhaonan19880921@126.com (N.Z.); ruizhao926@126.com (R.Z.); apolle@gwdg.de (A.P.); 2 Forestry Institute of New Technology, Chinese Academy of Forestry, Beijing 100091, China; 3 Guangdong Provincial Key Laboratory of Silviculture, Protection and Utilization, Guangdong Academy of Forestry, Guangzhou 510520, China; 4 Research Center of Saline and Alkali Land of National Forestry and Grassland Administration, Chinese Academy of Forestry, Beijing 100091, China; hlzhang2018@126.com; 5 Gansu Provincial Key Laboratory of Aridland Crop Science, Gansu Agricultural University, Lanzhou 730070, China; sag@gsau.edu.cn; 6 State Key Laboratory of Tree Genetics and Breeding, Research Institute of Forestry, Chinese Academy of Forestry, Beijing 100091, China; zhangyuhong512008@163.com; 7 Urat Desert-Grassland Research Station, Northwest Institute of Eco-Environment and Resources, Chinese Academy of Sciences, Lanzhou 730000, China; maxujun@lzb.ac.cn; 8 Forest Botany and Tree Physiology, University of Göttingen, 37077 Göttingen, Germany

**Keywords:** annexins, calcium-permeable channels, Cd flux, MAJ, NaCl, NAU, *Paxillus involutus*, *Populus* × *canescens*, PM H^+^-ATPase

## Abstract

Cadmium (Cd^2+^) pollution occurring in salt-affected soils has become an increasing environmental concern in the world. Fast-growing poplars have been widely utilized for phytoremediation of soil contaminating heavy metals (HMs). However, the woody Cd^2+^-hyperaccumulator, *Populus* × *canescens*, is relatively salt-sensitive and therefore cannot be directly used to remediate HMs from salt-affected soils. The aim of the present study was to testify whether colonization of *P.* × *canescens* with ectomycorrhizal (EM) fungi, a strategy known to enhance salt tolerance, provides an opportunity for affordable remediation of Cd^2+^-polluted saline soils. Ectomycorrhization with *Paxillus involutus* strains facilitated Cd^2+^ enrichment in *P.* × *canescens* upon CdCl_2_ exposures (50 μM, 30 min to 24 h). The fungus-stimulated Cd^2+^ in roots was significantly restricted by inhibitors of plasmalemma H^+^-ATPases and Ca^2+^-permeable channels (CaPCs), but stimulated by an activator of plasmalemma H^+^-ATPases. NaCl (100 mM) lowered the transient and steady-state Cd^2+^ influx in roots and fungal mycelia. Noteworthy, *P. involutus* colonization partly reverted the salt suppression of Cd^2+^ uptake in poplar roots. EM fungus colonization upregulated transcription of plasmalemma H^+^-ATPases (*PcHA4*, *8*, *11*) and annexins (*PcANN1*, *2*, *4*), which might mediate Cd^2+^ conductance through CaPCs. EM roots retained relatively highly expressed *PcHAs* and *PcANNs*, thus facilitating Cd^2+^ enrichment under co-occurring stress of cadmium and salinity. We conclude that ectomycorrhization of woody hyperaccumulator species such as poplar could improve phytoremediation of Cd^2+^ in salt-affected areas.

## 1. Introduction

Cadmium (Cd^2+^) pollution presents a critical threat to ecological environment and human life [1,2,3,4,5]. The Cd^2+^ contamination occurring in salt-affected soils has become an increasing environmental concern in recent years [6,7,8,9,10,11,12,13,14,15,16,17]. Coastal areas are polluted by Cd^2+^ due to rapid urbanization and industrialization. Cadmium is mainly derived from wastewater discharged by electroplating, mining, smelting, fuel, battery and chemical industry [18]. In some coastal saline zones, soil heavy metal pollution also comes from sludge and sewage irrigation [19]. Mining activities cause the release and spread of both hazardous heavy metals (HMs) and soluble salts in inland regions [11]. The Cd^2+^ contamination in salt-affected soils complicates remediation processes [6,7]. Naturally occurring halophytes may be potentially useful for remediation and phytomanagement [6,20,21,22,23]. However, halophytic species are commonly characterized by slow growth and therefore low biomass production [24]. Poplar trees have been widely utilized for phytoremediation of soils and water resources contaminated with HMs, because of their fast-growth, large biomass and remarkable Cd^2+^ accumulation in shoots and below-ground [25,26,27,28,29,30,31]. Moreover, several poplars, e.g., *Populus tremula*, *P.* × *canescens*, are known Cd^2+^ hyperaccumulators [32,33] in terms of the buildup of heavy metals in aerial parts (i.e., 100 times higher than non-accumulators) [34,35,36,37]. However, despite its high ability to tolerate Cd^2+^ stress [29,33,38], *P.* × *canescens* is relatively salt-sensitive [39] and therefore cannot be directly utilized to remediate HMs from salt-affected soils. The use of salt-resistant poplar, *P. euphratica*, is also hindered because this species is relatively susceptible to Cd^2+^ stress [40,41,42,43]. Therefore, efficient phytomanagement of heavy metal-contaminated salt soils with fast-growing poplars requires increased abilities of the plants to deal with the ionic stress situations produced by heavy metals and salts [6].

Ectomycorrhization offers great potential and feasibility for remediation of cadmium-contaminated soils [44,45,46,47,48,49,50]. Ectomycorrhization is the formation of symbiosis of a soil fungus with plant roots, whereby the root tip is completely ensheathed by the fungal hyphae. The plant benefits from this interaction by improved mineral nutrition and health [51]. Colonization of roots of *P.* × *canescens* with *Paxillus involutus*, an ectomycorrhizal (EM) fungus, has been repeatedly shown to improve Cd^2+^ uptake and tolerance [48,52]. The association of *Populus canadensis* with *P. involutus* leads to a highly significant increase of Cd^2+^ uptake and root-to-shoot transport, thus enhancing the total Cd^2+^ extraction by *P. canadensis* [44]. *P. involutus* ameliorates the negative effects of Cd^2+^ on shoot and root growth and chlorophyll content of old needles in Norway spruce seedlings (*Picea abies*) [53]. A protective effect against Cd^2+^ toxicity in the host was observed in *Pinus sylvestris* colonized with *P. involutus* [54,55]. *P. involutus* strains have also been used for phytoremediation of other heavy metals. Inoculation with a lead (Pb^2+^)-tolerant strain of *P. involutus* improves growth and Pb^2+^ tolerance of *P.* × *canescens* [56,57]. *P. involutus* decreases Pb^2+^ in roots and the translocation from the roots to the stems in Norway spruce *(Picea abies*) [58,59]. Similarly, *P. involutus* fungi act as a safety net that can immobilize large amounts of zinc, thus preventing transport to the host plant, *Pinus sylvestris* [60]. Moreover, ectomycorrhization of *P.* × *canescens* with *P. involutus* increases salt tolerance by maintaining nutrient uptake of K^+^, Ca^2+^ and NO_3_^−^, and improves Na^+^ homeostasis in the symbiotic associations [61,62,63,64,65,66]. Thus, it can be hypothesized that *P. involutus* could increase plant ability for Cd^2+^ enrichment in salt-affected soils. Arbuscular mycorrhizal fungi are able to enhance growth of pigeonpea (*Cajanus cajan*) by lowering Cd^2+^ content and strengthening antioxidant defense under NaCl and Cd stress [67]. Whether the ectomycorrhizal fungus *P.* *involutus* can mediate Cd^2+^ uptake under co-existing stress of NaCl and cadmium needs to be clarified by further experimental investigations.

Under cadmium stress, the *P. involutus*-facilitated Cd^2+^ influx is stimulated by plasma membrane (PM) H^+^-ATPases in EM roots [48]. Upregulated transcription of the PM H^+^-ATPase genes (*HA2.1* and *AHA10.1*) results in accelerated Cd^2+^ transport into roots of transgenic [38] and EM poplars [52]. Increased proton pumping activity and transcription of H^+^-ATPases have also been observed in EM *P.* × *canescens* under salt stress [66]. H^+^-ATPases maintain a proton gradient across PM to drive the entry of Cd^2+^ [38,48] and nutrient elements, such as K^+^, Ca^2+^, and NO_3_^−^, in addition to promotion of Na^+^/H^+^ antiport [64,65,66]. Moreover, the *P. involutus*-activated H^+^-pumps hyperpolarize the membrane potential, facilitating Cd^2+^ influx via hyperpolarization-activated Ca^2+^-permeable channels (CaPCs) [48]. Although the *P. involutus*-stimulated H^+^-ATPase enhances Cd^2+^ uptake under single stress of cadmium [48,52], little is known whether the fungi-activated H^+^-ATPase could improve Cd^2+^ enrichment in combined stress of CdCl_2_ and NaCl.

Cellular uptake of Cd^2+^ also involves the PM CaPCs, as demonstrated for various species [38,41,48,68]. Plant annexins (ANNs) might serve as channels to allow the entry of Ca^2+^ [69,70,71,72,73,74,75,76] or indirectly mediate Ca^2+^ conductance [77,78]. Chen et al. suggested that OsANN4 mediates the transmembrane Cd^2+^ influx along rice roots [73]. The *P. euphratica* annexin ANN1 facilitates Cd^2+^ enrichment through CaPCs in roots of transgenic Arabidopsis [79]. *P.* × *canescens* colonization with *P. involutus* leads to Cd^2+^ enrichment [52] due to stimulation of Cd^2+^ influx via CaPCs [48]. Cadmium treatment results in increased transcript levels of annexins in maize (*ZmAnx9*, [80]), peanut (*ANNAh3*, [81]), and rice (*ANN4*, [73]). Whether *P*. × *canescens* annexins are affected by cadmium and contribute to Cd^2+^ enrichment in *P. involutus* ectomycorrhizal associations needs to be investigated. Under sodium chloride salinity, competition between Na^+^ and Cd^2+^ for Ca^2+^ ion channels reduced Cd^2+^ uptake in *Amaranthus mangostanus* [82]. The salt effects on annexin-mediated Ca^2+^ channels remain unclear in ectomycorrhizal roots under co-existing stress conditions of Cd^2+^ and NaCl.

In this study, we examined the impact of ectomycorrhizal fungi on root Cd^2+^ uptake under combined stress of salt and cadmium, aiming to elucidate the underlying mechanisms. We used two different *P. involutus* isolates, MAJ and NAU, for this study. Strain MAJ forms a complete ectomycorrhiza composed of a thick hyphal mantle ensheathing root tip and a typical Hartig net structure inside the roots for nutrient exchange, while strain NAU forms only the outer mantle [83]. We studied Cd^2+^ uptake in the presence and absence of NaCl and analyzed gene expression of annexins because previous studies show that PeANN1 facilitates Cd^2+^ enrichment through CaPCs [79]. *P. involutus* activates H^+^-pumps and hyperpolarizes membrane potential in EM roots [48,64,65]. Therefore, the PM H^+^-ATPases-promoted Cd^2+^ flux was also verified in EM roots under salt stress. Our data reveal that *P. involutus* inoculation stimulates Cd^2+^ influx under salt stress, resulting from the upregulated H^+^-ATPases and annexins in the ectomycorrhizal roots. Both MAJ and NAU conserved the Cd^2+^ uptake capacities under co-occurring stresses of cadmium and salinity, regardless of the formation of Hartig net in the ectomycorrhizal symbioses.

## 2. Results

### 2.1. Cd^2+^ Concentrations in Roots and Shoots of Ectomycorrhizal Poplars under NaCl Stress

**Figure 1:**
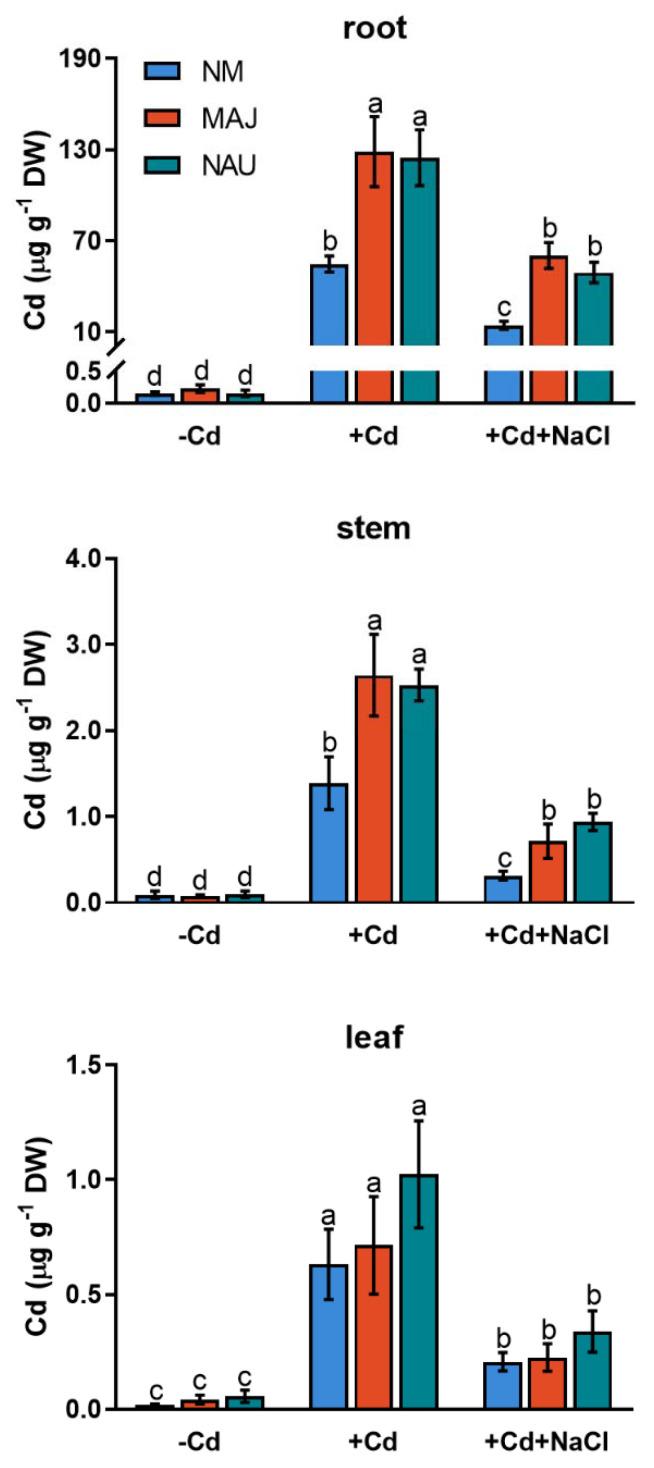
Cd^2+^ concentrations in roots, stems and leaves of non-mycorrhizal (NM) and ectomycorrhizal (EM) *Populus × canescens* under cadmium and salt stress. Poplar plantlets inoculated with or without *Paxillus involutus* isolates (MAJ or NAU, 30 d), were hydroponically acclimated and subjected to 24 h of CdCl_2_ (0 or 50 μM) in combination with NaCl (0 or 100 mM). Mean values of Cd^2+^ concentrations in control (−Cd), CdCl_2_ stress (+Cd), and combined stress of CdCl_2_ and NaCl (+Cd + NaCl) are shown. Each column is mean ± SD obtained from 3 individual plants. Statistically significant differences (*p* < 0.05) among treatments are indicated with different letters (a–d).

Cd^2+^ concentrations were analyzed in roots, stems and leaves of NM and EM *P*. × *canescens* after 24 h exposure to CdCl_2_ (50 µM) or combined stress of CdCl_2_ and NaCl (100 mM). Under CdCl_2_ stress, non-mycorrhizal (NM) roots displayed remarkably higher Cd^2+^ concentrations than stem and leaves (Figure 1). Compared to NM plants, Cd^2+^ concentrations were 0.8- to 1.4-fold higher in roots and stems of poplars colonized with *P. involutus* isolates, MAJ and NAU (Figure 1). However, the addition of NaCl (100 mM) significantly decreased Cd^2+^ accumulation in roots and shoots of both NM- and EM-plants (Figure 1). Of note, EM-plants retained significantly higher Cd^2+^ concentrations in roots and stems than NM poplars under salt stress (Figure 1). Therefore, EM fungi enhanced Cd^2+^ enrichment in both root and aerial parts of *P. × canescens* under co-occurring stresses of cadmium and salinity.

### 2.2. Steady-State Cd^2+^ Influx in Ectomycorrhizal Poplar Roots and Fungal Mycelia under NaCl Stress

**Figure 2:**
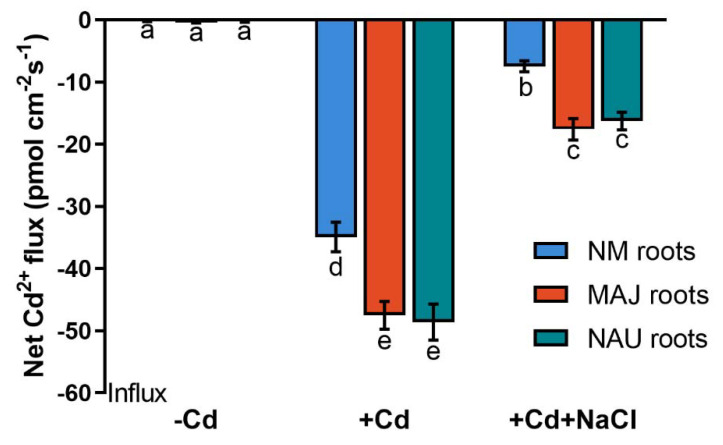
Steady-state Cd^2+^ fluxes in non-mycorrhizal (NM) *Populus × canescens* and ectomycorrhizal (EM) roots under cadmium and salt stress. Poplar plantlets inoculated with or without *Paxillus involutus* isolates (MAJ or NAU, 30 d), were hydroponically acclimated and subjected to 24 h of CdCl_2_ (0 or 50 μM) in combination with NaCl (0 or 100 mM). Root tips were excised from EM- and NM-poplars and equilibrated for 30 min in measuring solution. Net fluxes of Cd^2+^ along root axis (100 to 2300 μm) were monitored at an interval of 200–300 μm (Figure S1). Mean values of Cd^2+^ fluxes in control (−Cd), CdCl_2_ stress (+Cd), and combined stress of CdCl_2_ and NaCl (+Cd + NaCl) are shown. Cd^2+^ flux was not detectable in salt controls that were treated without CdCl_2_. Each column is mean ± SD obtained from 5 individual plants. Statistically significant differences (*p* < 0.05) among treatments are indicated with different letters (a–e).

To determine whether the Cd^2+^ enrichment in EM *P.* × *canescens* resulted from the *P. involutus*-stimulated uptake, Cd^2+^ fluxes were examined in NM-, EM-roots and fungal mycelia under CdCl_2_ and NaCl stress. CdCl_2_ exposure (50 µM, 24 h) resulted in an apparent Cd^2+^ uptake, 34.9 pmol cm^−2^ s^−1^, along NM-roots of the hyperaccumulator, *Populus* × *canescens* (Figure 2 and Figure S1). EM-roots exhibited 36% to 39% higher Cd^2+^ fluxes than the NM-roots (Figure 2). The presence of NaCl (100 mM) significantly decreased the flux rates in both NM- and EM-roots but the EM-roots still exhibited 1.2–1.4-fold greater Cd^2+^ uptake than the NM-roots (Figure 2 and Figure S1). The effect of NaCl on root Cd^2+^ fluxes resembles the trend of Cd^2+^ accumulation in salinized NM- and EM-roots (Figure 1 and Figure 2).

**Figure 3:**
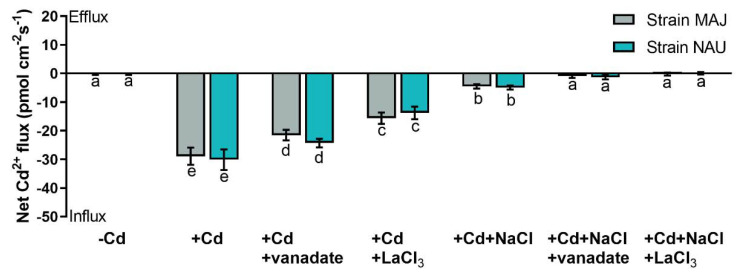
Net Cd^2+^ fluxes in fungal hyphae of *Paxillus involutus* isolates (MAJ and NAU) under cadmium, salt, and inhibitor treatments. MAJ and NAU mycelia (the youngest and active hyphae) were hydroponically acclimated and subjected to 24 h of CdCl_2_ (0 or 50 μM) in combination with NaCl (0 or 100 mM). The short-term Cd- and Cd + NaCl-stressed fungal mycelia were treated with an inhibitor of plasmalemma H^+^-ATPase (sodium orthovanadate, 0 or 500 μM) or an inhibitor of Ca^2+^-permeable channels (LaCl_3_, 0 or 5 mM) for 30 min. Following 30 min equilibration in measuring solutions, Cd^2+^ flux recordings were continued for 15 min on the surface of pelleted hyphae. Mean values of Cd^2+^ fluxes in control (−Cd), CdCl_2_ stress (+Cd), and combined stress of CdCl_2_ and NaCl (+Cd + NaCl) in the presence and absence of inhibitors are shown. Cd^2+^ flux was not detectable in salt controls that were treated without CdCl_2_. Each column is mean ± SD obtained from 5 fungal cultures. Statistically significant differences (*p* < 0.05) among treatments are indicated with different letters (a–e).

Fungal hyphae of the two tested *P. involutus* isolates, MAJ and NAU, showed a drastic Cd^2+^ influx, 28.9–30.1 pmol cm^−2^ s^−1^, under CdCl_2_ treatment (50 µM, 24 h, Figure 3). NaCl reduced the Cd^2+^ influx by 84–85% in the mycelia (Figure 3), which is similar to the reduction in EM-roots upon salinity stress (Figure 2).

### 2.3. Transient Cd^2+^ Kinetics and Membrane Potential upon Salt Shock

**Figure 4:**
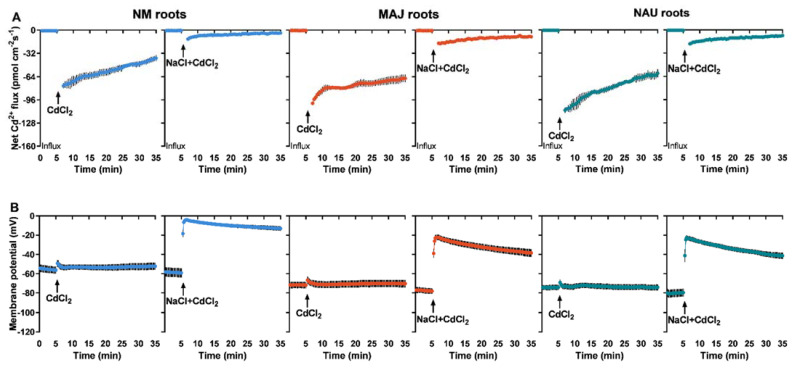
CdCl_2_ and NaCl shock-altered Cd^2+^ kinetics and membrane potential in non-mycorrhizal (NM) *Populus* × *canescens* and ectomycorrhizal (EM) roots. (**A**) Cd^2+^ flux kinetics. (**B**) Membrane potential. Poplar plantlets were inoculated with or without *Paxillus involutus* isolates (MAJ or NAU) for 30 d. Root tips were excised from EM- and NM-poplars and equilibrated for 30 min in Cd^2+^ or H^+^ measuring solution. At the apical zones Cd^2+^ fluxes and membrane potential were recorded before and after the addition of CdCl_2_ (100 μM) or a combined solution of CdCl_2_ (50 μM) and NaCl (100 mM). The recordings continued respectively for 5 and 30 min before and after the cadmium and salt shock. Each data point is mean ± SD obtained from 5 individual plants.

CdCl_2_ shock (50 µM) created a transient Cd^2+^ influx in roots of NM *P.* × *canescens*, although the flux gradually decreased with prolonged exposure time (Figure 4A). EM-roots exhibited a pattern similar to NM-roots but with typically higher influx rates (Figure 4A). The Cd^2+^ influxes in both NM- and EM-roots were markedly reduced upon the NaCl addition (Figure 4A), similar to reduction found for the steady-state Cd^2+^ influx in salinized roots (Figure 2). Compared with the EM-roots, the restriction effect of NaCl was more pronounced in NM-roots (Figure 4A).

Transient kinetics of membrane potential upon CdCl_2_ (50 µM) and NaCl (100 mM) shocks were compared between roots of NM- and EM-poplars because the membrane potential indicates activity of PM H^+^-ATPase [66]. NMT recordings showed that the resting membrane potential ranged from −54.4 to −59.2 mV in NM-roots under control conditions (Figure 4B). EM-roots had a more strongly hyperpolarized PM, with a membrane potential ranging from −71.7 to −80.8 mV (Figure 4B). CdCl_2_ shock exerted no significant effects on the membrane potential in NM- and EM-roots, although a marginal rise (5.0–6.1 mV) was observed after the onset of CdCl_2_ addition, which returned to the pretreatment level 1–2 min after Cd^2+^ addition (Figure 4B). However, the addition of NaCl together with CdCl_2_ caused an immediate and substantial depolarization of the membrane potential in NM- and EM-roots, although the PM tended to be rehyperpolarized during prolonged exposure to NaCl + CdCl_2_ (Figure 4B). In comparison, the membrane potential in EM-roots was less depolarized (−22.2 to −41.4 mV) after the onset of CdCl_2_ + NaCl shock as compared to NM-roots (−4.1 to −13.0 mV, Figure 4B). 

### 2.4. Effects of PM H^+^-ATPase Inhibitor and Activator on Cd^2+^ Uptake

**Figure 5:**
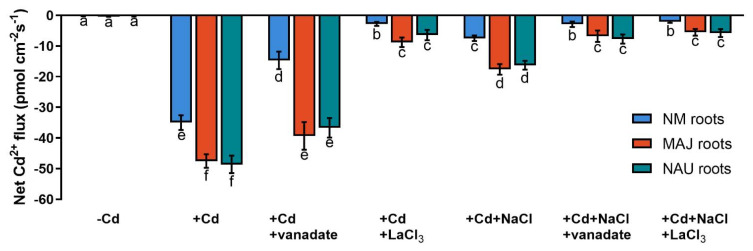
Net Cd^2+^ fluxes in non-mycorrhizal (NM) *Populus* × *canescens* and ectomycorrhizal (EM) roots under cadmium, salt, and inhibitor treatments. Poplar plantlets inoculated with or without *Paxillus involutus* isolates (MAJ or NAU, 30 d), were hydroponically acclimated and subjected to 24 h of CdCl_2_ (0 or 50 μM) in combination with NaCl (0 or 100 mM). Root tips were excised from EM- and NM-poplars and subjected to an inhibitor of plasmalemma H^+^-ATPase (sodium orthovanadate, 0 or 500 μM) or an inhibitor of Ca^2+^-permeable channels (LaCl_3_, 0 or 5 mM) for 30 min. Following 30 min equilibration in measuring solutions, net fluxes of Cd along root axis (100 to 2300 μm) were monitored at an interval of 200–300 μm (Figures S2 and S3). Mean values of Cd^2+^ fluxes in control (−Cd), CdCl_2_ stress (+Cd), and combined stress of CdCl_2_ and NaCl (+Cd + NaCl) in the presence and absence of inhibitors are shown. Cd^2+^ flux was not detectable in salt controls that were treated without CdCl_2_. Each column is mean ± SD obtained from 5 individual plants. Statistically significant differences (*p* < 0.05) among treatments are indicated with different letters (a–f).

Cd^2+^ transport in poplar trees is accelerated by the PM H^+^-ATPase [38,48,52]. An H^+^-pump inhibitor, orthovanadate, was used to testify the crucial role of H^+^-pumps for Cd^2+^ uptake in NM-, EM-roots, and fungal hyphae under CdCl_2_ and salt stress. In NM-roots, orthovanadate decreased the Cd^2+^ influx approximately two-fold, while in EM-roots only 17–25% decreases were found (Figure 5 and Figure S2). In mycelia, vanadate also caused moderately reduced Cd^2+^ influx (Figure 3). In the presence of NaCl, the inhibition of orthovanadate was evident in the fungus and roots, although the Cd^2+^ influx had been significantly lowered by the salt treatment (Figure 3, Figure 5, and Figure S2).

**Figure 6:**
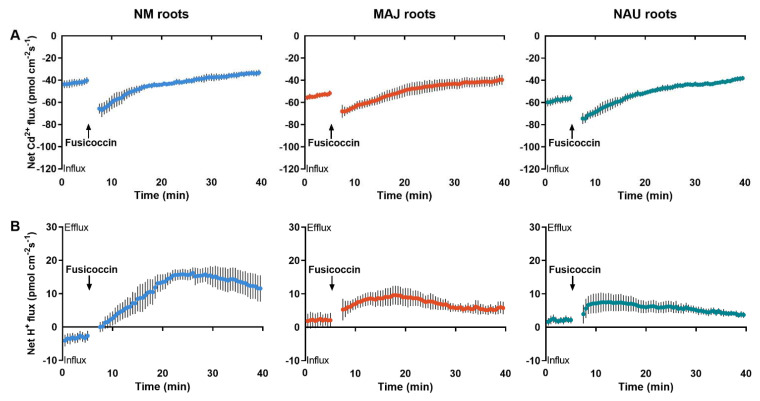
Fusicoccin shock-altered Cd^2+^ and H^+^ kinetics in non-mycorrhizal (NM) *Populus* × *canescens* and ectomycorrhizal (EM) roots. (**A**) Cd^2+^ flux kinetics. (**B**) H^+^ flux kinetics. Poplar plantlets inoculated with or without *Paxillus involutus* isolates (MAJ or NAU, 30 d) were hydroponically acclimated and subjected to 24 h of CdCl_2_ (50 μM). Root tips were excised from EM- and NM-poplars and equilibrated for 30 min in Cd^2+^ or H^+^ measuring solution. At the apical zones, Cd^2+^ and H^+^ fluxes were recorded before and after the addition of fusicoccin (10 μM). The recordings continued, respectively, for 5 and 35 min before and after fusicoccin shock. Each data point is mean ± SD obtained from 5 individual plants.

Furthermore, the activator of PM H^+^-ATPase, fusicoccin (FC), was used to test the effect of H^+^ pumping on Cd^2+^ uptake in short-term stressed roots. Following the CdCl_2_ treatment (50 µM, 24 h), roots of NM- and EM-poplars were subjected to FC activation. Immediately after the onset of FC addition, a stimulation of Cd^2+^ influxes was observed at the surface of NM- and EM-roots (Figure 6A). H^+^ efflux was correspondingly increased in FC-treated NM- and EM-roots (Figure 6B), indicating that H^+^ pumps were transiently activated [84,85,86,87]. The observation that the increase in H^+^ efflux corresponded to the Cd^2+^ influx in *P.* × *canescens* roots suggests that the uptake of Cd^2+^ was promoted by the H^+^-ATPases in the PM.

### 2.5. Transcriptional Activation of H^+^-ATPase in Ectomycorrhizal P. × canescens

**Figure 7:**
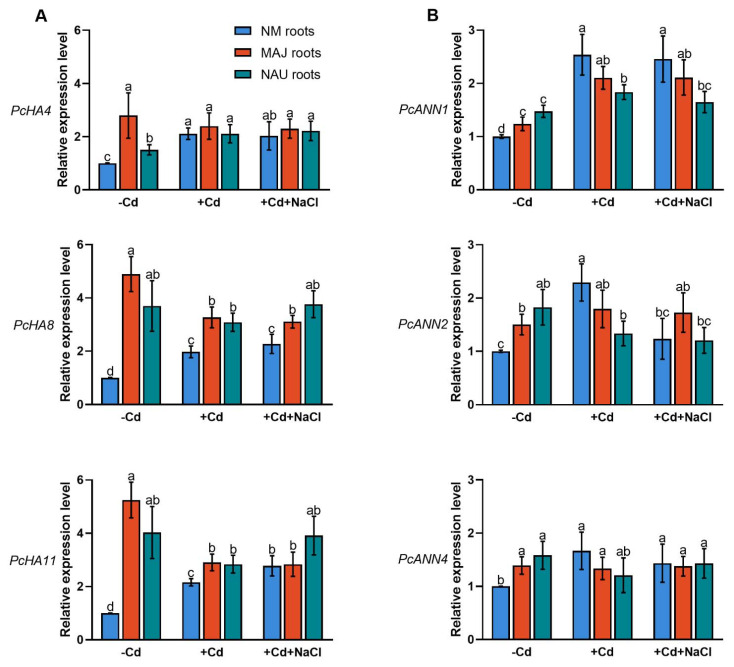
Effects of CdCl_2_ as single stress factor or in combination with NaCl on transcriptional profiles of plasmalemma H^+^-ATPase (*PcHAs*) and annexins (*PcANNs*) in roots of non-mycorrhizal or ectomycorrhizal (EM) *Populus* × *canescens.* (**A**) *PcHA4*, *8*, *11*. (**B**) *PcANN1*, *2*, *4*. Poplar plantlets inoculated with or without *Paxillus involutus* isolates (MAJ or NAU, 30 d), were hydroponically acclimated and subjected to 24 h of CdCl_2_ (0 or 50 μM) in combination with NaCl (0 or 100 mM). Roots were harvested from EM- and NM-poplars and used for total RNA isolation and RT-qPCR. *18S* *rRNA* was used as a reference gene. Specific primers designed to target *PcHA4, 8, 11*, *PcANN1, 2, 4* and *18S* *rRNA* are shown in Table S1. Mean values of *PcHAs* and *PcANNs* relative transcript levels in control (−Cd), CdCl_2_ stress (+Cd), and combined CdCl_2_ and NaCl stress (+Cd + NaCl) are shown. Each column is mean ± SD obtained from 3 independent experiments. Statistically significant differences (*p* < 0.05) among treatments are indicated with different letters (a–d).

Transcript levels of the PM H^+^-ATPase-encoding genes, *PcHA4*, *PcHA8* and *PcHA11*, were examined in NM and EM roots since these three *PcHAs* were previously shown to be differently expressed under control and Na^+^ stress conditions [66]. EM-roots showed significantly higher (0.5–4.2 fold) transcript levels of *PcHA4*, *PcHA8* and *PcHA11* than NM-roots (Figure 7A). This observation agrees with Sa et al. (2019) [66]. Cadmium treatment (50 µM CdCl_2_, 24 h) resulted in upregulation of *PcHA4*, *PcHA8*, and *PcHA11* in NM-roots (Figure 7A). In contrast, Cd^2+^ caused a 14–45% decline of *PcHAs* in EM-roots, with the exception of *PcHA4* in NAU roots (Figure 7A). It is notable that the transcript levels, in particular those of *PcHA8*, and *PcHA11,* still remained higher in the EM- than in NM-roots, despite the decline caused by Cd^2+^ stress (Figure 7A). 

NaCl treatment (100 mM, 24 h) lowers the transcript levels of *PcHAs* (*4, 8, 11*) in NM-roots [66]. Here, exposure to NaCl of the Cd^2+^-treated roots did not result in decreased *PcHA4* and *PcHA8* transcript levels and an increase of *PcHA11* was observed (Figure 7A). Similarly, NaCl did not significantly change *PcHAs* transcription in EM-roots in the presence of Cd^2+^ (Figure 7A). We noticed that EM-roots retained overall higher transcript levels of *PcHAs* than NM-roots under co-occurring stresses of cadmium and salinity.

### 2.6. Calcium Channel Inhibitor Blocks Cd^2+^ Fluxes

Cadmium ions enter the plasma membrane through CaPCs in plant cells [48,79,88]. To determine whether CaPCs contributed to the mediation of Cd^2+^ influx under combined CdCl_2_ and NaCl stress, LaCl_3_ was used to block Ca^2+^-channels in the roots of NM- and EM-poplars. The inhibitor significantly decreased root Cd^2+^ uptake in the presence and absence of NaCl, although NaCl treatment reduced the apparent Cd^2+^ influx under coexisting stress (Figure 5 and Figure S3). Similarly, the LaCl_3_ significantly reduced Cd^2+^ uptake in fungal hyphae regardless of the NaCl addition (Figure 3).

### 2.7. Transcript Levels of Annexin Genes in Ectomycorrhizal P. × canescens

Plant annexins (ANNs), such as ANN1, ANN2, ANN4, function as Ca^2+^-permeable channels in higher plants [70,71,72,73,74,75,76,79,89]. We have shown that *P. euphratica* PeANN1 facilitates cadmium enrichment by regulation of calcium-permeable channels [79]. Here, we examined the *P.* × *canescens* orthologs *PcANN1*, *PcANN2* and *PcANN4* in NM- and EM-roots. In the absence of Cd and salt, *PcANN1*, *PcANN2* and *PcANN4* showed significantly higher transcripts in EM-roots than in the NM (Figure 7B). This observation is in accord with previous findings that EM-roots retain typically higher influx of Ca^2+^ than NM-roots [64,65]. Short-term cadmium exposure (50 µM, 24 h) caused significant increases of *PcANN* transcript levels in NM roots (Figure 7B), supporting Cd^2+^ enrichment in the woody hyperaccumulator [29,33,38,52]. The Cd^2+^ stimulation of annexin transcript levels was less pronounced in EM-roots (Figure 7B). For example, *PcANN1* levels which increased by 25–70% in MAJ and NAU roots under Cd^2+^ treatment were still lower than those in CdCl_2_-treated NM-roots (Figure 7B). The *PcANN2* responded differently to short-term cadmium exposure in the EM-roots colonized with the strain MAJ (increase) and the strain NAU (decrease) (Figure 7B). Cadmium exposure also slightly decreased *PcANN4* in EM-roots (4–24%, Figure 7B). In CdCl_2_-stressed NM roots, NaCl lowered the transcripts of *PcANNs* by 3–46% (Figure 7B). As a result, the cadmium stimulation of annexin genes (with the exception of *PcANN1*) was lost by the addition of NaCl (Figure 7B). Compared to NM-roots, *PcANNs* was either less (*PcANN1, PcANN2*) or not reduced (*PcANN4*) by NaCl in EM-roots under cadmium treatment (Figure 7B).

## 3. Discussion

### 3.1. The P. Involutus-Activated PM H^+^-ATPase Contributes to Cd^2+^ Enrichment in EM Roots

Our data show that the woody hyperaccumulator, *P.* × *canescens*, exhibited strong Cd^2+^ uptake and accumulation in root and shoots, which is further enhanced by colonizing with EM-fungus *P. involutus* (Figure 1). These findings are similar to previous reports in long-term studies [29,33,48,52]. The root flux recordings confirmed that the enhanced Cd^2+^ entry in *P.* × *canescens* roots was due to the colonization with MAJ and NAU isolates, which were characterized by a remarkable Cd^2+^ enrichment in the hyphae (Figure 2, Figure 3, and Figure S1) [48,90]. However, we observed that salt stress caused by NaCl reduced the Cd^2+^ influx in roots and fungus (Figure 2, Figure 3, and Figure S1). Similarly, NaCl reduced root cadmium uptake and translocation in the halophyte *Carpobrotus rossii* [7,8] and *Atriplex halimus* [91]. An important novel result was that the *P. involutus* could alleviate the salt suppression of Cd^2+^ uptake in *P.* × *canescens* roots (Figure 2, Figure 4, and Figure S1). To obtain a mechanistic understanding of the underlying processes, we inhibited and stimulated the Cd^2+^ fluxes with pharmacological agents. The entry of Cd^2+^ in the roots and fungal hyphae declined when the plasmalemma H^+^-ATPase was inhibited by vanadate (Figure 3, Figure 5, and Figure S2) [48] and increased when the plasmalemma H^+^-ATPase was stimulated by FC (Figure 6). These data suggest that Cd^2+^ uptake required a proton gradient [48,52]. Moreover, *P. involutus* colonization resulted in a higher H^+^ efflux and correspondingly a more negative membrane potential (Figure 6), indicating that the PM H^+^-ATPases were activated by the ectomycorrhiza [48,64,66]. This is similar to the enhanced proton-ATPase in arbuscular-mycorrhizal symbiosis [92,93]. The highly activated H^+^-pumps hyperpolarize the PM, thereby facilitating Cd^2+^ influx via hyperpolarization-activated CaPCs [48,73]. In accordance with our flux analyses, transcript levels of the PM H^+^-ATPase-encoding genes, *PcHA4*, *PcHA8*, *PcHA11*, generally remained at higher levels in ectomycorrhizal roots under control and CdCl_2_ stress compared to NM *P.* × *canescens* roots, although two or three of the tested *PcHAs* were down-regulated by CdCl_2_ in MAJ and NAU roots (Figure 7). Of note, EM-roots maintained higher transcripts of *PcHA4*, *8*, *11* than non-colonized roots under combined stress of CdCl_2_ and NaCl (Figure 7). Similarly, Sa et al. showed that both MAJ and NAU roots retain higher transcript levels of *PcHA4* and/or *PcHA8* than NM-roots under control and NaCl stress conditions [66]. Increased abundances of PM H^+^-ATPase transcripts are expected to contribute to the activated H^+^-pumps because the plasmalemma H^+^-ATPases are transcriptionally regulated in poplars [85,86,94]. Thus, the retained H^+^-pumping activity resulted in less depolarization of membrane potential under NaCl stress (Figure 4) [66], thereby upkeeping Cd^2+^ influx into the EM-roots. This result concurs with those of Ma et al. (2014), who found that upregulation of *HA2.1* and *AHA10.1* leads to Cd^2+^ uptake in EM poplar roots [52].

### 3.2. The Fungus-Elicited Annexins Mediated Cd^2+^ Uptake in EM Roots

Since LaCl_3_ inhibited Cd^2+^ uptake into roots and fungal hyphae, our results support that Cd^2+^ uptake involves CaPCs in the PM (Figure 3, Figure 5, and Figure S3) [41,48,68,73,88]. Plant annexins, in particular ANN1, ANN2 and ANN4, have been shown to function as CaPCs in Arabidopsis, maize and rice [70,71,72,73,75,76]. Zhang et al. suggested that PeANN1 facilitates the flow of cadmium ions through CaPCs [79]. CdCl_2_ treatment upregulated transcripts of *PcANN1*, *PcANN2* and *PcANN4* in roots of NM *P.* × *canescens* (Figure 7), similar to the findings in crop species, such as maize, peanut and rice [73,80,81]. Accordingly, the cadmium-elicited annexins might mediate root Cd^2+^ inflow through CaPCs in the poplar, contributing to its hyperaccumulator character [33]. Noteworthy, *PcANN1*, *PcANN2* and *PcANN4* showed remarkably higher transcripts in EM-roots than in the non-colonized under control conditions; CdCl_2_ treatment caused a further increase in *PcANN1* in EM-roots and *PcANN2* was specifically increased in MAJ-colonized roots (Figure 7). The arbuscular mycorrhiza-stimulated transcription of *GmAnn1a* was observed in soybean roots [95]. In addition, annexin proteins also showed enhanced accumulation in arbuscular mycorrhizal roots of *Medicago sativa* and *M. truncatula* following cadmium application [96,97]. Therefore, the fungus-induced annexins might have collectively contributed to the CaPCs-mediated Cd^2+^ enrichment in root cells of the poplar [73,79]. In accordance with this notion, we have previously shown that *Paxillus*-colonized roots showed higher Ca^2+^ and Cd^2+^ influxes than NM-roots [48,64,65]. We noticed that the transcripts of *PcANN1, 2, 4* in EM-roots exhibited lower levels than non-colonized roots under CdCl_2_ stress (Figure 7). However, root Cd^2+^ influx remained higher in EM than in NM (Figure 2 and Figure 4). Thus, it can be inferred that the annexin-mediated uptake of Cd^2+^ was mainly promoted by the electrochemical gradient across the PM that was established by H^+^-ATPases. NaCl decreased *PcANN2* and *PcANN4* in NM-roots, but the transcript levels of *PcANN1*, *2, 4* were less reduced by NaCl in EM-roots (Figure 7). It is worth noting that in these ectomycorrhizal roots, transcription of *PcHAs* was retained at high levels under cadmium and salinity stress (Figure 7). Taken together, this suggests that the fungus-stimulated transcription of *annexins* contributed to Cd^2+^ enrichment in EM-roots under combined stresses of cadmium and salt.

## 4. Materials and Methods

### 4.1. Fungal Inoculation with Populus × canescens

The two isolates of EM fungus *P. involutus* (MAJ and NAU) from Büsgen-Institute: Forest Botany and Tree Physiology (Göttingen University, Büsgenweg 2, Göttingen, Germany) were cultured on modified Melin Norkrans medium [83]. *P.* × *canescens* plantlets were micropropagated and rooted in modified Murashige and Skoog (MMS) medium [98]. Uniform and healthy plantlets were inoculated with MAJ or NAU for 30 d using a Petri-dish culture system [99].

### 4.2. Cadmium and NaCl Treatment

The agar plugs with hyphae, and plants colonized with or without EM fungus, were hydroponically acclimated in MMS nutrient solution for 2–3 d [66]. Then fungal mycelia, NM- and EM-plants were treated with CdCl_2_ (0 or 50 μM) in combination with NaCl (0 or 100 mM) in MMS solution. Following 24 h of CdCl_2_ treatment and combined stress of CdCl_2_ and NaCl, steady-state Cd^2+^ fluxes were recorded in fungal mycelia, NM- and EM-roots. Transcript levels of genes encoding annexins (*PcANN1, 2, 4*) and PM H^+^-ATPases (*PcHA4, 8, 11*) were examined in control and stressed roots.

### 4.3. Inhibitor and Activator Treatment

The fungal mycelia, NM-, and EM-roots pretreated with short-term CdCl_2_ or CdCl_2_ + NaCl were exposed to inhibitors of Ca^2+^ channels (LaCl_3_, 0 or 5 mM) [48,100] or PM H^+^-ATPases (sodium orthovanadate, 0 or 500 μM) [100,101] for 30 min. Steady-state Cd^2+^ fluxes were recorded on the surface of roots and pelleted hyphae, respectively [48].

After 24 h exposure to 50 μM CdCl_2_, roots from NM- and EM-poplars were subjected to an activator of PM H^+^-ATPase, Fusicoccin (FC). FC produced by *Fusicoccum amygdali*, has the function of activating H^+^-ATPase in the PM [102,103]. Cd^2+^ and H^+^ transient kinetics were continuously recorded for 35 min after FC (10 μM) were added to measuring solutions.

### 4.4. Assessed of Cd^2+^ Concentrations

After 24 h exposure to CdCl_2_ (0 or 50 μM) in combination with NaCl (0 or 100 mM), roots, stems and leaves of NM- and EM-poplars were sampled and oven dried at 70–80 °C for 5 d. Dried samples was weighed 0.1 g and digested in 5 mL of concentrated HNO_3_ and 2 mL 30% H_2_O_2_ in a microwave accelerated reaction system (Titan MPS Microwave Sample Preparation System, Perkin-Elmer, Waltham, MA, USA). Concentrations of Cd^2+^ were assessed by a PerkinElmer Optima 8000 ICP-OES Spectrometer (Perkin-Elmer, Waltham, MA, USA).

### 4.5. Flux Recordings of Cd^2+^ and H^+^

#### 4.5.1. Microelectrodes Preparation and Calibration

Cd^2+^ and H^+^ flux profiles were recorded using an NMT system (NMT-YG-100, Younger USA LLC, Amherst, MA, USA). The glass microelectrodes were prepared as previously described [42,43,48,84,104]. Prior to flux recordings, the calibration of Cd^2+^- and H^+^-selective microelectrodes were carried out in the following standards (concentrations in mM):

(a) H^+^ microelectrodes: 0.1 NaCl, 0.1 CaCl_2_, 0.1 MgCl_2_, and 0.5 KCl, pH 4.5, 5.5, and 6.5 (pH was adjusted to 5.3 during H^+^ flux recordings); and
(b) Cd^2+^ microelectrodes: 0.05 CaCl_2_, 0.1 MgCl_2_, 0.5 KCl, 0 or 100 NaCl, and CdCl_2_ series (0.01, 0.05, and 0.1), pH 5.3 (Cd^2+^ concentration was 0.05 mM during Cd^2+^ flux recordings).

After calibration, the microelectrodes that showed Nernstian slopes of 58 ± 6 mV/decade (H^+^) and 29 ± 4 mV/decade (Cd^2+^) were used in our NMT recordings.

#### 4.5.2. Steady-State Cd^2+^ Flux Recordings

After 24 h exposure to CdCl_2_ (0 or 50 μM) in combination with NaCl (0 or 100 mM), sodium orthovanadate (0 or 500 μM), and LaCl_3_ (0 or 5 mM), fungal mycelia and root tips excised from NM- and EM-poplars were subjected to 30 min equilibration in the following measuring solutions (concentrations in mM), respectively:

(i) Control (−Cd): 0.05 CaCl_2_, 0.1 MgCl_2_, 0.5 KCl, pH 5.3;
(ii) +Cd: 0.05 CaCl_2_, 0.05 CdCl_2_, 0.1 MgCl_2_, 0.5 KCl, pH 5.3; and
(iii) Cd+NaCl: 0.05 CaCl_2_, 0.05 CdCl_2_, 0.1 MgCl_2_, 0.5 KCl, 100 NaCl, pH 5.3.

Following equilibration, net fluxes of Cd^2+^ along root axis (100 to 2300 μm) were monitored at an interval of 200–300 μm. The flux recording at each point was continued for 6–8 min [41,64,101,105]. For the fungal mycelia, Cd^2+^ flux recording of pelleted hyphae was continued 15 min [48]. Cd^2+^ fluxes were recorded from at least five individual plants or fungal cultures for each treatment. The flux oscillations in EM fungus and poplars are not so pronounced as that observed in crop seedlings [48,106].

#### 4.5.3. Transient Recordings of Cd^2+^, H^+^ Flux and Membrane Potential

Transient Cd^2+^ Kinetics and Membrane Potential.

NM- and EM-roots were incubated in basic solutions of Cd^2+^ (concentration in mM: 0.05 CaCl_2_, 0.1 MgCl_2_, 0.5 KCl, pH 5.3) and H^+^ (0.1 CaCl_2_, 0.1 MgCl_2_, 0.1 NaCl, 0.5 KCl, pH 5.3) for 30 min. Cd^2+^ fluxes and membrane potentials at apical regions were recorded for 5 min prior to CdCl_2_ and NaCl shocks. Membrane potential was measured using Ag/AgCl microelectrodes (XY-CGQ03; Xuyue (Beijing) Sci and Tech Co. Ltd., Suzhou street 49, Haidian District, Beijing, China) as previously described [66]. Then, CdCl_2_ (100 μM) stock, or a combined stock solution of CdCl_2_ (100 μM) and NaCl (200 mM) was added slowly to reach final concentrations of 50 μM (CdCl_2_) and 100 mM (NaCl). Kinetics of membrane potential and Cd^2+^ uptake were recorded up to 30 min in NM- and EM-roots. Cd^2+^ fluxes and membrane potentials were recorded from at least five individual plants for each treatment.

Transient Kinetics of Cd^2+^ and H^+^ upon FC.

The NM- and EM-roots pretreated with CdCl_2_ (50 μM, 24 h) were excised and equilibrated in measuring solutions of Cd^2+^ or H^+^ for 30 min. Fluxes of Cd^2+^ and H^+^ at apical regions were recorded for 5 min before the addition of FC (Sigma-Aldrich, St. Louis, MO, USA). Then, FC stock solution (dissolved in DMSO) was added to Cd^2+^ and H^+^ measuring solutions, reaching a final concentration of 10 μM [103]. Cd^2+^ and H^+^ transient kinetics in FC-treated roots were further recorded for 35 min. Fluxes of Cd^2+^ and H^+^ were recorded from at least five individual plants for NM-, MAJ- and NAU-roots.

### 4.6. Determination of Gene Expression of Annexins and PM H^+^-ATPases 

After 24 h exposure to CdCl_2_ (0 or 50 μM), or to CdCl_2_ (50 μM) in combination with NaCl (100 mM), total RNA was isolated from NM and fungus-colonized roots and used for real-time quantitative PCR (RT-qPCR) [66]. The primer sequences for annexins (*PcANN1, 2, 4*) [79], plasmalemma H^+^ ATPase (*PcHAs, PcHA4*, *8*, *11*) [66], and reference genes (*18S rRNA*) [107], are shown in Table S1. The RT-qPCR amplification was performed as previously described [66,79,86]. Expression profiles for *PcANNs* and *PcHAs* were normalized to the transcripts of *18S rRNA* [108]. The RT-qPCR experiment was repeated three times.

### 4.7. Data Analysis

The calculations of flux rate and membrane potential were processed using JCal V3.2.1 program (Xuyue (Beijing) Sci and Tech Co. Ltd., Suzhou street 49, Haidian District, Beijing, China, Available online: http://www.xuyue.net/, accessed on 12 March 2021). All experimental data were subjected to SPSS version 19.0 (IBM Corporation, Armonk, NY, USA). Differences between means were considered significant at *p* < 0.05.

## 5. Conclusions

Our data provide further evidence that cadmium can be enriched in ectomycorrhizal poplars under co-existing stress conditions of Cd^2+^ and NaCl. *P. involutus* stimulated Cd^2+^ influx through CaPCs in ectomycorrhizal *P.* × *canescens* roots, depending on the plasmalemma H^+^-ATPase. NaCl lowered the uptake of Cd^2+^ in poplar roots, which was alleviated by ectomycorrhization with *P. involutus*. Ectomycorrhizal fungus colonization upregulated transcription of PM H^+^-ATPases (*PcHA4, 8, 11*) and increased transcripts of annexins (*PcANN1, 2, 4*), which might mediate Cd^2+^ conductance through PM CaPCs. NaCl-treated EM-roots retained relatively highly expressed *PcHAs* and *PcANNs*. We hypothesize that the sustained transcription of *PcHAs* resulted in H^+^ pumping activity and PM hyperpolarization in the ectomycorrhiza, thus promoting Cd^2+^ enrichment through the PcANNs-mediated Ca^2+^ channels in EM-roots under co-occurring stresses of cadmium and salinity. Although the colonization of MAJ and NAU varies with regard to the formation of intraradical hyphae, i.e., the Hartig net, both strains conserved higher Cd^2+^ uptake under salt stress than NM-roots. We propose that *P. involutus* strains, which have been repeatedly shown to improve salt tolerance, may be applied as beneficial microbes to improve plant phytoremediation for cadmium in salt-affected areas.

## Data Availability

The data presented in this study are available in the article and Supplementary Materials.

## Supplementary Materials

The following are available online at https://www.mdpi.com/article/10.3390/ijms222111651/s1.

## References

[1] Nawrot T., Plusquin M., Hogervorst J., Roels H.A., Celis H., Thijs L., Vangronsveld J., Van Hecke E., Staessen J.A. (2006). Environmental exposure to cadmium and risk of cancer: A prospective population-based study. Lancet Oncol..

[2] Krämer U. (2010). Metal hyperaccumulation in plants. Annu. Rev. Plant Biol..

[3] Kaplan O., Ince M., Yaman M. (2011). Sequential extraction of cadmium in different soil phases and plant parts from a former industrialized area. Environ. Chem. Lett..

[4] Luo Z.B., He J.L., Polle A., Rennenberg H. (2016). Heavy metal accumulation and signal transduction in herbaceous and woody plants: Paving the way for enhancing phytoremediation efficiency. Biotechnol. Adv..

[5] Shi W.G., Zhang Y.H., Chen S.L., Polle A., Rennenberg H., Luo Z.B. (2019). Physiological and molecular mechanisms of heavy metal accumulation in nonmycorrhizal versus mycorrhizal plants. Plant Cell Environ..

[6] Lutts S., Lefèvre I. (2015). How can we take advantage of halophyte properties to cope with heavy metal toxicity in salt-affected areas?. Ann. Bot..

[7] Cheng M.M., Kopittke P.M., Wang A.A., Tang C.X. (2018). Salinity decreases Cd translocation by altering Cd speciation in the halophytic Cd-accumulator *Carpobrotus rossii*. Ann. Bot..

[8] Cheng M.M., Wang A.A., Liu Z.Q., Gendall A.R., Rochfort S., Tang C.X. (2018). Sodium chloride decreases cadmium accumulation and changes the response of metabolites to cadmium stress in the halophyte *Carpobrotus rossii*. Ann. Bot..

[9] Guo S.H., Jiang L.Y., Xu Z.M., Li Q.S., Wang J.F., Ye H.J., Wang L.L., He B.Y., Zhou C., Zeng E.Y. (2020). Biological mechanisms of cadmium accumulation in edible Amaranth (*Amaranthus mangostanus* L.) cultivars promoted by salinity: A transcriptome analysis. Environ. Pollut..

[10] Hao L.T., Chen L.H., Zhu P., Zhang J., Zhang D.J., Xiao J.J., Xu Z.F., Zhang L., Liu Y., Li H. (2020). Sex-specific responses of *Populus deltoides* to interaction of cadmium and salinity in root systems. Ecotoxicol. Environ. Saf..

[11] Khan A., Bilal S., Khan A.L., Imran M., Al-Harrasi A., Al-Rawahi A., Lee I.J. (2020). Silicon-mediated alleviation of combined salinity and cadmium stress in date palm (*Phoenix dactylifera* L.) by regulating physio-hormonal alteration. Ecotoxicol. Environ. Saf..

[12] Nosek M., Kaczmarczyk A., Jędrzejczyk R.J., Supel P., Kaszycki P., Miszalski Z. (2020). Expression of genes involved in heavy metal trafficking in plants exposed to salinity stress and elevated Cd concentrations. Plants.

[13] Pastuszak J., Kopeć P., Płażek A., Gondek K., Szczerba A., Hornyák M., Dubert F. (2020). Cadmium accumulation in the grain of durum wheat is associated with salinity resistance degree. Plant Soil Environ..

[14] Wiszniewska A., Kamińska I., Hanus-Fajerska E., Sliwinska E., Koźmińska A. (2020). Distinct co-tolerance responses to combined salinity and cadmium exposure in metallicolous and non-metallicolous ecotypes of *Silene vulgaris*. Ecotoxicol. Environ. Saf..

[15] Zhang S.L., Ni X.L., Arif M., Yuan Z.X., Li L.J., Li C.X. (2020). Salinity influences Cd accumulation and distribution characteristics in two contrasting halophytes, *Suaeda glauca* and *Limonium aureum*. Ecotoxicol. Environ. Saf..

[16] Zhang S.L., Ni X.L., Arif M., Zheng J., Stubbs A., Li C.X. (2020). NaCl improved Cd tolerance of the euhalophyte *Suaeda glauca* but not the recretohalophyte *Limonium aureum*. Plant Soil.

[17] Zhu Q.L., Bao J.J., Liu J.H., Zheng J.L. (2020). High salinity acclimatization alleviated cadmium toxicity in *Dunaliella salina*: Transcriptomic and physiological evidence. Aquat. Toxicol..

[18] Benavides M.P., Gallego S.M., Tomaro M.L. (2005). Cadmium toxicity in plants. Braz. J. Plant Physiol..

[19] Mao J.H., Shen W.R. (2005). Reflection of soil salination pollution research and landuse of Binhai area in Tianjin. Tianjin Agric. Sci..

[20] Amari T., Ghnaya T., Debez A., Taamali M., Youssef N.B., Lucchini G., Sacchi G.A., Abdelly C. (2014). Comparative Ni tolerance and accumulation potentials between *Mesembryanthemum crystallinum* (halophyte) and *Brassica juncea*: Metal accumulation, nutrient status and photosynthetic activity. J. Plant Physiol..

[21] Wang H.L., Tian C.Y., Jiang L., Wang L. (2014). Remediation of heavy metals contaminated saline soils: A halophyte choice?. Environ. Sci. Technol..

[22] Liang L.C., Liu W.T., Sun Y.B., Huo X.H., Li S., Zhou Q.X. (2016). Phytoremediation of heavy metal contaminated saline soils using halophytes: Current progress and future perspectives. Environ. Rev..

[23] Nikalje G.C., Suprasanna P. (2018). Coping with metal toxicity–cues from halophytes. Front. Plant Sci..

[24] Masters D.G., Norman H.C., Khan M.A., Ozturk M., Gul B., Ahmed M.Z. (2016). 15—Genetic and Environmental Management of Halophytes for Improved Livestock Production. Halophytes for Food Security in Dry Lands.

[25] Schützendübel A., Nikolova P., Rudolf C., Polle A. (2002). Cadmium and H_2_O_2_-induced oxidative stress in *Populus* × *canescens* roots. Plant Physiol. Bioch..

[26] Rockwood D.L., Naidu C.V., Carter D.R., Rahmani M., Spriggs T.A., Lin C., Alker G.R., Isebrands J.G., Segrest S.A., Nair P.K.R., Rao M.R., Buck L.E. (2004). Short-rotation woody crops and phytoremediation: Opportunities for agroforestry?. New Vistas in Agroforestry.

[27] Unterbrunner R., Puschenreiter M., Sommer P., Wieshammer G., Tlustoš P., Zupan M., Wenzel W.W. (2007). Heavy metal accumulation in trees growing on contaminated sites in Central Europe. Environ. Pollut..

[28] Di Lonardo S., Capuana M., Arnetoli M., Gabbrielli R., Gonnelli C. (2011). Exploring the metal phytoremediation potential of three *Populus alba* L. clones using an in vitro screening. Environ. Sci. Pollut. Res..

[29] He J.L., Li H., Luo J., Ma C.F., Li S.J., Qu L., Gai Y., Jiang X.N., Janz D., Polle A. (2013). A transcriptomic network underlies microstructural and physiological responses to cadmium in *Populus* × *canescens*. Plant Physiol..

[30] He J.L., Ma C.F., Ma Y.L., Li H., Kang J.Q., Liu T.X., Polle A., Peng C.H., Luo Z.B. (2013). Cadmium tolerance in six poplar species. Environ. Sci. Pollut. Res..

[31] Pajević S., Borišev M., Nikolić N., Arsenov D.D., Orlović S., Župunski M., Ansari A., Gill S., Gill R., Lanza G., Newman L. (2016). Phytoextraction of heavy metals by fast-growing trees: A review. Phytoremediation.

[32] Kieffer P., Planchon S., Oufir M., Ziebel J., Dommes J., Hoffmann L., Hausman J.F., Renaut J. (2009). Combining proteomics and metabolite analyses to unravel cadmium stress-response in poplar leaves. J. Proteome Res..

[33] He J.L., Qin J.J., Long L.Y., Ma Y.L., Li H., Li K., Jiang X.N., Liu T.X., Polle A., Liang Z.S. (2011). Net cadmium flux and accumulation reveal tissue-specific oxidative stress and detoxification in *Populus* × *canescens*. Physiol. Plant..

[34] Brooks R.R., Lee J., Reeves R.D., Jaffré T. (1977). Detection of nickeliferous rocks by analysis of herbarium specimens of indicator plants. J. Geochem. Explor..

[35] Brooks R.R., Brooks R.R. (1998). Phytochemistry of hyperaccumulators. Plants That Hyperaccumulate Heavy Metals: Their Role in Phytoremediation, Microbiology, Archaeology, Mineral Exploration, and Phytomining.

[36] Chaney R.L., Malik M., Li Y.M., Brown S.L., Brewer E.P., Angle J.S., Baker A.J. (1997). Phytoremediation of soil metals. Curr. Opin. Biotechnol..

[37] Salt D.E., Smith R.D., Raskin I. (1998). Phytoremediation. Annu. Rev. Plant Physiol. Plant Mol. Biol..

[38] He J.L., Li H., Ma C.F., Zhang Y.L., Polle A., Rennenberg H., Cheng X.Q., Luo Z.B. (2015). Overexpression of bacterial *γ*-glutamylcysteine synthetase mediates changes in cadmium influx, allocation and detoxification in poplar. New Phytol..

[39] Janz D., Behnke K., Schnitzler J.P., Kanawati B., Schmitt-Kopplin P., Polle A. (2010). Pathway analysis of the transcriptome and metabolome of salt sensitive and tolerant poplar species reveals evolutionary adaption of stress tolerance mechanisms. BMC Plant Biol..

[40] Polle A., Klein T., Kettner C. (2013). Impact of cadmium on young plants of *Populus euphratica* and *P.* × *canescens*, two poplar species that differ in stress tolerance. New Forest..

[41] Sun J., Wang R.G., Zhang X., Yu Y.C., Zhao R., Li Z.Y., Chen S.L. (2013). Hydrogen sulfide alleviates cadmium toxicity through regulations of cadmium transport across the plasma and vacuolar membranes in *Populus euphratica* cells. Plant Physiol. Bioch..

[42] Han Y.S., Sa G., Sun J., Shen Z.D., Zhao R., Ding M.Q., Deng S.R., Lu Y.J., Zhang Y.H., Shen X. (2014). Overexpression of *Populus euphratica* xyloglucan endotransglucosylase/hydrolase gene confers enhanced cadmium tolerance by the restriction of root cadmium uptake in transgenic tobacco. Environ. Exp. Bot..

[43] Han Y.S., Wang S.J., Zhao N., Deng S.R., Zhao C.J., Li N.F., Sun J., Zhao R., Yi H.L., Shen X. (2016). Exogenous abscisic acid alleviates cadmium toxicity by restricting Cd^2+^ influx in *Populus euphratica* cells. J. Plant Growth Regul..

[44] Sell J., Kayser A., Schulin R., Brunner I. (2005). Contribution of ectomycorrhizal fungi to cadmium uptake of poplars and willows from a heavily polluted soil. Plant Soil.

[45] Krpata D., Peintner U., Langer I., Fitz W.J., Schweiger P. (2008). Ectomycorrhizal communities associated with *Populus tremula* growing on a heavy metal contaminated site. Mycol. Res..

[46] Krpata D., Fitz W., Peintner U., Langer I., Schweiger P. (2009). Bioconcentration of zinc and cadmium in ectomycorrhizal fungi and associated aspen trees as affected by level of pollution. Environ. Pollut..

[47] Luo Z.B., Wu C.H., Zhang C., Li H., Lipka U., Polle A. (2014). The role of ectomycorrhizas in heavy metal stress tolerance of host plants. Environ. Exp. Bot..

[48] Zhang Y.H., Sa G., Zhang Y.N., Zhu Z.M., Deng S.R., Sun J., Li N.F., Li J., Yao J., Zhao N. (2017). *Paxillus involutus*-facilitated Cd^2+^ influx through plasma membrane Ca^2+^-permeable channels is stimulated by H_2_O_2_ and H^+^-ATPase in ectomycorrhizal *Populus* × *canescens* under cadmium stress. Front. Plant Sci..

[49] Tang Y., Shi L., Zhong K., Shen Z., Chen Y. (2019). Ectomycorrhizal fungi may not act as a barrier inhibiting host plant absorption of heavy metals. Chemosphere.

[50] Hachani C., Lamhamedi M.S., Cameselle C., Gouveia S., Zine El Abidine A., Khasa D.P., Béjaoui Z. (2020). Effects of Ectomycorrhizal Fungi and Heavy Metals (Pb, Zn, and Cd) on Growth and Mineral Nutrition of *Pinus halepensis* Seedlings in North Africa. Microorganisms.

[51] Dreischhoff S., Das I.S., Jakobi M., Kasper K., Polle A. (2020). Local responses and systemic induced resistance mediated by ectomycorrhizal fungi. Front. Plant Sci..

[52] Ma Y.L., He J.L., Ma C.F., Luo J., Li H., Liu T.X., Polle A., Peng C.H., Luo Z.B. (2014). Ectomycorrhizas with *Paxillus involutus* enhance cadmium uptake and tolerance in *Populus* × *canescens*. Plant Cell Environ..

[53] Jentschke G., Winter S., Godbold D.L. (1999). Ectomycorrhizas and cadmium toxicity in Norway spruce seedlings. Tree Physiol..

[54] Colpaert J.V., Vanassche J.A. (1993). The effects of cadmium on ectomycorrhizal *Pinus sylvestris* L. New Phytol..

[55] Hartley-Whitaker J., Cairney J.W.G., Meharg A.A. (2000). Sensitivity to Cd or Zn of host and symbiont of ectomycorrhizal *Pinus sylvestris* L. (Scots pine) seedlings. Plant Soil.

[56] Szuba A., Karliński L., Krzesłowska M., Hazubska-Przybył T. (2017). Inoculation with a Pb-tolerant strain of *Paxillus involutus* improves growth and Pb tolerance of *Populus* × *canescens* under *in vitro* conditions. Plant. Soil.

[57] Szuba A., Marczak Ł., Kozłowski R. (2020). Role of the proteome in providing phenotypic stability in control and ectomycorrhizal poplar plants exposed to chronic mild Pb stress. Environ. Pollut..

[58] Marschner P., Godbold D.L., Jentschke G. (1996). Dynamics of lead accumulation in mycorrhizal and non-mycorrhizal Norway spruce (*Picea abies* (L) Karst). Plant Soil.

[59] Jentschke G., Marschner P., Vodnik D., Marth C., Bredemeier M., Rapp C., Fritz E., Gogala N., Godbold D.L. (1998). Lead uptake by *Picea abies* seedlings: Effects of nitrogen source and mycorrhizas. J. Plant Physiol..

[60] Colpaert J.V., Vanassche J.A. (1992). Zinc toxicity in ectomycorrhizal *Pinus sylvestris*. Plant Soil.

[61] Langenfeld-Heyser R., Gao J., Ducic T., Tachd P., Lu C.F., Fritz E., Gafur A., Polle A. (2007). *Paxillus involutus* mycorrhiza attenuate NaCl-stress responses in the salt-sensitive hybrid poplar *Populus* × *canescens*. Mycorrhiza.

[62] Luo Z.B., Janz D., Jiang X.N., Göbel C., Wildhagen H., Tan Y.P., Rennenberg H., Feussner I., Polle A. (2009). Upgrading root physiology for stress tolerance by ectomycorrhizas: Insights from metabolite and transcriptional profiling into reprogramming for stress anticipation. Plant Physiol..

[63] Luo Z.B., Li K., Gai Y., Göbel C., Wildhagen H., Jiang X.N., Feußner I., Rennenberg H., Polle A. (2011). The ectomycorrhizal fungus (*Paxillus involutus*) modulates leaf physiology of poplar towards improved salt tolerance. Environ. Exp. Bot..

[64] Li J., Bao S.Q., Zhang Y.H., Ma X.J., Mishra-Knyrim M., Sun J., Sa G., Shen X., Polle A., Chen S.L. (2012). *Paxillus involutus* strains MAJ and NAU mediate K^+^/Na^+^ homeostasis in ectomycorrhizal *Populus* × *canescens* under sodium chloride stress. Plant Physiol..

[65] Ma X.J., Sun M., Sa G., Zhang Y.H., Li J., Sun J., Shen X., Polle A., Chen S.L. (2014). Ion fluxes in *Paxillus involutus*-inoculated roots of *Populus* × *canescens* under saline stress. Environ. Exp. Bot..

[66] Sa G., Yao J., Deng C., Liu J., Zhang Y.N., Zhu Z.M., Zhang Y.H., Ma X.J., Zhao R., Lin S.Z. (2019). Amelioration of nitrate uptake under salt stress by ectomycorrhiza with and without a Hartig net. New Phytol..

[67] Garg N., Chandel S. (2015). Role of arbuscular mycorrhiza in arresting reactive oxygen species (ROS) and strengthening antioxidant defense in *Cajanus cajan* (L.) Millsp. nodules under salinity (NaCl) and cadmium (Cd) stress. Plant Growth Regul..

[68] Li L.Z., Liu X.L., Peijnenburg W.J., Zhao J.M., Chen X.B., Yu J.B., Wu H.F. (2012). Pathways of cadmium fluxes in the root of the halophyte *Suaeda salsa*. Ecotoxicol. Environ. Saf..

[69] Hofmann A., Proust J., Dorowski A., Schantz R., Huber R. (2000). Annexin 24 from *Capsicum annuum*: X-ray structure and biochemical characterization. J. Biol. Chem..

[70] Laohavisit A., Mortimer J.C., Demidchik V., Coxon K.M., Stancombe M.A., Macpherson N., Brownlee C., Hofmann A., Webb A.A., Miedema H. (2009). *Zea mays* Annexins modulate cytosolic free Ca^2+^ and generate a Ca^2+^-permeable conductance. Plant Cell.

[71] Laohavisit A., Shang Z.L., Rubio L., Cuin T.A., Véry A.A., Wang A.H., Mortimer J.C., Macpherson N., Coxon K.M., Battey N.H. (2012). *Arabidopsis* annexin1 mediates the radical-activated plasma membrane Ca^2+^- and K^+^-permeable conductance in root cells. Plant Cell.

[72] Liao C.C., Zheng Y., Guo Y. (2017). MYB30 transcription factor regulates oxidative and heat stress responses through ANNEXIN-mediated cytosolic calcium signaling in *Arabidopsis*. New Phytol..

[73] Chen X.H., Ouyang Y.N., Fan Y.C., Qiu B.Y., Zhang G.P., Zeng F.R. (2018). The pathway of transmembrane cadmium influx via calcium-permeable channels and its spatial characteristics along rice root. J. Exp. Bot..

[74] Demidchik V., Shabala S., Isayenkov S., Cuin T.A., Pottosin I. (2018). Calcium transport across plant membranes: Mechanisms and functions. New Phytol..

[75] Ma L., Ye J.M., Yang Y.Q., Lin H.X., Yue L.L., Luo J., Long Y., Fu H.Q., Liu X.N., Zhang Y.L. (2019). The SOS2-SCaBP8 Complex Generates and Fine-Tunes an AtANN4-Dependent Calcium Signature under Salt Stress. Dev. Cell.

[76] Liu Q.B., Ding Y.L., Shi Y.T., Ma L., Wang Y., Song C.P., Wilkins K.A., Davies J.M., Knight H., Knight M.R. (2021). The calcium transporter ANNEXIN1 mediates cold-induced calcium signaling and freezing tolerance in plants. EMBO J..

[77] Konopka-Postupolska D., Clark G., Hofmann A. (2011). Structure, function and membrane interactions of plant annexins: An update. Plant Sci..

[78] Konopka-Postupolska D., Clark G. (2017). Annexins as overlooked regulators of membrane trafficking in plant cells. Int. J. Mol. Sci..

[79] Zhang Y.N., Sa G., Zhang Y., Hou S.Y., Wu X., Zhao N., Zhang Y.H., Deng S.R., Deng C., Deng J.Y. (2021). *Populus euphratica* annexin1 facilitates cadmium enrichment in transgenic Arabidopsis. J. Hazard. Mater..

[80] Zhou M.L., Yang X.B., Zhang Q., Zhou M., Zhao E.Z., Tang Y.X., Zhu X.M., Shao J.R., Wu Y.M. (2013). Induction of annexin by heavy metals and jasmonic acid in *Zea mays*. Funct. Integr. Genom..

[81] He M.J., Yang X.L., Cui S.L., Mu G.J., Hou M.Y., Chen H.Y., Liu L.F. (2015). Molecular cloning and characterization of annexin genes in peanut (*Arachis hypogaea* L.). Gene.

[82] Mei X., Li S., Li Q., Yang Y., Luo X., He B., Li H., Xu Z. (2014). Sodium chloride salinity reduces Cd uptake by edible amaranth (*Amaranthus mangostanus* L.) via competition for Ca channels. Ecotoxicol. Environ. Saf..

[83] Gafur A., Schützendübel A., Langenfeld-Heyser R., Fritz E., Polle A. (2004). Compatible and incompetent *Paxillus involutus* isolates for ectomycorrhiza formation *in vitro* with poplar (*Populus* × *canescens*) differ in H_2_O_2_ production. Plant Biol..

[84] Sun J., Chen S.L., Dai S.X., Wang R.G., Li N.Y., Shen X., Zhou X.Y., Lu C.F., Zheng X.J., Hu Z.M. (2009). NaCl-induced alternations of cellular and tissue ion fluxes in roots of salt-resistant and salt-sensitive poplar species. Plant Physiol..

[85] Wang M.J., Wang Y., Sun J., Ding M.Q., Deng S.R., Hou P.C., Ma X.J., Zhang Y.H., Wang F.F., Sa G. (2013). Overexpression of *PeHA1* enhances hydrogen peroxide signaling in salt-stressed *Arabidopsis*. Plant Physiol. Biochem..

[86] Yao J., Shen Z.D., Zhang Y.L., Wu X., Wang J.H., Sa G., Zhang Y.H., Zhang H.L., Deng C., Liu J. (2020). *Populus euphratica* WRKY1 binds the promoter of H^+^-ATPase gene to enhance gene expression and salt tolerance. J. Exp. Bot..

[87] Zhang H.L., Deng C., Wu X., Yao J., Zhang Y.L., Zhang Y.N., Deng S.R., Zhao N., Zhao R., Zhou X.Y. (2020). *Populus euphratica* remorin 6.5 activates plasma membrane H^+^-ATPases to mediate salt tolerance. Tree Physiol..

[88] Perfus-Barbeoch L., Leonhardt N., Vavasseur A., Forestier C. (2002). Heavy metal toxicity: Cadmium permeates through calcium channels and disturbs the plant water status. Plant J..

[89] Laohavisit A., Brown A.T., Cicuta P., Davies J.M. (2010). Annexins: Components of the calcium and reactive oxygen signaling network. Plant Physiol..

[90] Ott T., Fritz E., Polle A., Schützendübel A. (2002). Characterisation of antioxidative systems in the ectomycorrhiza-building basidiomycete *Paxillus involutus* (Bartsch) Fr. and its reaction to cadmium. FEMS Microbiol. Ecol..

[91] Lefèvre I., Marchal G., Meerts P., Corréal E., Lutts S. (2009). Chloride salinity reduces cadmium accumulation by the Mediterranean halophyte species *Atriplex halimus* L. Environ. Exp. Bot..

[92] Ramos A.C., Martins M.A., Façanha A.R. (2005). ATPase and pyrophosphatase activities in corn root microsomes colonized with arbuscular mycorrhizal fungi. Braz. J. Plant Physiol..

[93] Rosewarne G.M., Smith F.A., Schachtman D.P., Smith S.E. (2007). Localization of proton-ATPase genes expressed in arbuscular mycorrhizal tomato plants. Mycorrhiza.

[94] Ding M.Q., Hou P.C., Shen X., Wang M.J., Deng S.R., Sun J., Xiao F., Wang R.G., Zhou X.Y., Lu C.F. (2010). Salt-induced expression of genes related to Na^+^/K^+^ and ROS homeostasis in leaves of salt-resistant and salt-sensitive poplar species. Plant Mol. Biol..

[95] Schaarschmidt S., Gresshoff P.M., Hause B. (2013). Analyzing the soybean transcriptome during autoregulation of mycorrhization identifies the transcription factors GmNF-YA1a/b as positive regulators of arbuscular mycorrhization. Genome Biol..

[96] Repetto O., Bestel-Corre G., Dumas-Gaudot E., Berta G., Gianinazzi-Pearson V., Gianinazzi S. (2003). Targeted proteomics to identify cadmium-induced protein modifications in *Glomus mosseae*-inoculated pea roots. New Phytol..

[97] Aloui A., Recorbet G., Gollotte A., Robert F., Valot B., Gianinazzi-Pearson V., Aschi-Smiti S., Dumas-Gaudot E. (2009). On the mechanisms of cadmium stress alleviation in *Medicago truncatula* by arbuscular mycorrhizal symbiosis: A root proteomic study. Proteomics.

[98] Leple J.C., Brasileiro A.C.M., Michel M.F., Delmotte F., Jouanin L. (1992). Transgenic poplars: Expression of chimeric genes using four different constructs. Plant Cell Rep..

[99] Müller A., Volmer K., Mishra-Knyrim M., Polle A. (2013). Growing poplars for research with and without mycorrhizas. Front. Plant Sci..

[100] Sun J., Wang M.J., Ding M.Q., Deng S.R., Liu M.Q., Lu C.F., Zhou X.Y., Shen X., Zheng X.J., Zhang Z.K. (2010). H_2_O_2_ and cytosolic Ca^2+^ signals triggered by the PM H^+^-coupled transport system mediate K^+^/Na^+^ homeostasis in NaCl-stressed *Populus euphratica* cells. Plant Cell Environ..

[101] Lu Y.J., Li N.Y., Sun J., Hou P.C., Jing X.S., Zhu H.P., Deng S.R., Han Y.S., Huang X.X., Ma X.J. (2013). Exogenous hydrogen peroxide, nitric oxide and calcium mediate root ion fluxes in two non-secretor mangrove species subjected to NaCl stress. Tree Physiol..

[102] De Boer B. (1997). Fusicoccin—A key to multiple 14-3-3 locks?. Trends Plant Sci..

[103] Kinoshita T., Shimazaki K.I. (2001). Analysis of the phosphorylation level in guard-cell plasma membrane H^+^-ATPase in response to fusicoccin. Plant Cell Physiol..

[104] Sun J., Dai S.X., Wang R.G., Chen S.L., Li N.Y., Zhou X.Y., Lu C.F., Shen X., Zheng X.J., Hu Z.M. (2009). Calcium mediates root K^+^/Na^+^ homeostasis in poplar species differing in salt tolerance. Tree Physiol..

[105] Sun J., Wang R.G., Liu Z.Q., Ding Y.Z., Li T.Q. (2013). Non-invasive microelectrode cadmium flux measurements reveal the spatial characteristics and real-time kinetics of cadmium transport in hyperaccumulator and nonhyperaccumulator ecotypes of *Sedum alfredii*. J. Plant Physiol..

[106] Shabala S., Shabala L., Gradmann D., Chen Z., Newman I., Mancuso S. (2006). Oscillations in plant membrane transport: Model predictions, experimental validation, and physiological implications. J. Exp. Bot..

[107] Junghans U., Polle A., Düchting P., Weiler E., Kuhlman B., Gruber F., Teichmann T. (2006). Adaptation to high salinity in poplar involves changes in xylem anatomy and auxin physiology. Plant Cell Environ..

[108] Livak K.J., Schmittgen T.D. (2001). Analysis of relative gene expression data using real-time quantitative PCR and the 2^−ΔΔCT^ method. Methods.

