# Supplementary material for: Ectomycorrhizal Fungal Strains Facilitate Cd2+ Enrichment in a Woody Hyperaccumulator under Co-Existing Stress of Cadmium and Salt
Source: Int J Mol Sci. 2021 Oct 28;22(21):11651. doi: 10.3390/ijms222111651 (PMC8583747; doi:10.3390/ijms222111651)
Supplement: Supplementary file 1 [file ijms-22-11651-s001.zip › Figure S3.pdf]

International Journal of
Molecular Sciences

MDPI

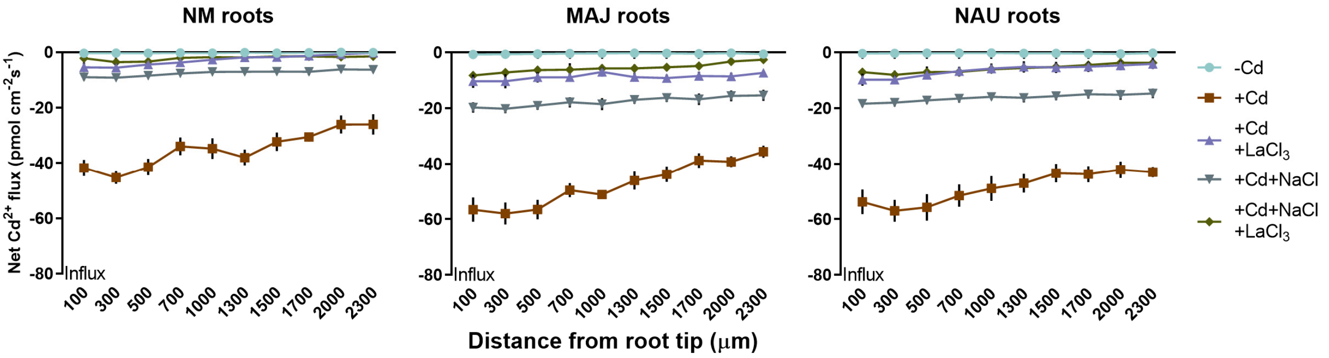

Figure S3. Effects of calcium channel inhibitors ($LaCl_3$) on steady-state fluxes of Cd^{2+} in *Populus×canescens* roots colonized without (nonmycorrhizal, NM) or with *Paxillus involutus* strains MAJ and NAU under salt stress. *P.* × *canescens* roots were inoculated without or with the *P. involutus* strains, MAJ and NAU, for 30 d, respectively. NM and fungus-colonized *P.* × *canescens* plants were exposed to $CdCl_2$ (0 or 50 μM) and NaCl (0 or 100 mM) for 24 h in modified MS nutrient solution. The short-term Cd- and NaCl-stressed roots were treated with $LaCl_3$ (0 or 5 mM) for 30 min. Following 30 min equilibration in specific measuring solutions, Cd^{2+} fluxes were measured along root axis, 100–2300 μm from the apex, at intervals of 200–300 μm. Cd^{2+} fluxes in control (−Cd), $CdCl_2$ stress (+Cd), and co-existing stress of $CdCl_2$ and NaCl (+Cd+NaCl) in the presence and absence of inhibitor are shown. Cd^{2+} flux was not detectable in salt controls that treated without $CdCl_2$. Each data point is mean ± SD obtained from 5 individual plants.
