# Supplementary material for: Ectomycorrhizal Fungal Strains Facilitate Cd2+ Enrichment in a Woody Hyperaccumulator under Co-Existing Stress of Cadmium and Salt
Source: Int J Mol Sci. 2021 Oct 28;22(21):11651. doi: 10.3390/ijms222111651 (PMC8583747; doi:10.3390/ijms222111651)
Supplement: Supplementary file 1 [file ijms-22-11651-s001.zip › Table S1.pdf]

International Journal of
Molecular Sciences

MDPI

Table S1. Gene-specific primers used for real-time (RT) quantitative PCR

Target	*Populus trichocarpa* Homolog locus	Primer name	Primer sequence (5'-3')
18S rRNA	*	Forward Primer	AGAAACGGCTACCACATCCAA
		Reverse Primer	CCAGACTTGCCCTCCAATGG
PcHA4	Potri.018G006000	Forward Primer	TTACGCCAAACTTATCTG
		Reverse Primer	CCTAAATGACGGAACAAT
PcHA8	Potri.018G112400	Forward Primer	AGCAAGAATCCCAGCACA
		Reverse Primer	CAGCATTGAAGAAGGCAGAC
PcHA11	Potri.012G071600	Forward Primer	CTGATTGAGTTCGGTGAA
		Reverse Primer	TATCCGCTATGCTTTGAG
PcANN1	Potri.002G095600	Forward Primer	CATCAGGATTTTGGCTACCAG
		Reverse Primer	CTCATCATTAGGATCGGCTT
PcANN2	Potri.007G092500	Forward Primer	AAGTGCTTGACCTACCCTGAA
		Reverse Primer	GCCCTGGTGGTTACAACTCTA
PcANN4	Potri.001G024900	Forward Primer	AGAAGCAAGGCTCATCTCAAA
		Reverse Primer	TCCACATCTGCTCGGGTC

* Junghans, U.; Polle, A.; Düchting, P.; Weiler, E.; Kuhlman, B.; Gruber, F.; Teichmann, T. Adaptation to high salinity in poplar involves changes in xylem anatomy and auxin physiology. *Plant. Cell Environ.* **2006**, *29*, 1519–1531. [107].
